# Chemical Composition and Antioxidant Properties of Powders Obtained from Different Plum Juice Formulations

**DOI:** 10.3390/ijms18010176

**Published:** 2017-01-17

**Authors:** Anna Michalska, Aneta Wojdyło, Grzegorz P. Łysiak, Adam Figiel

**Affiliations:** 1 Institute of Agricultural Engineering, Wrocław University of Environmental and Life Sciences, 51-630 Wrocław, Poland; adam.figiel@up.wroc.pl; 2 Department of Fruit, Vegetable and Cereals Technology, Wrocław University of Environmental and Life Sciences, 51-630 Wrocław, Poland; aneta.wojdylo@up.wroc.pl; 3 Poznań University of Life Sciences, 60-594 Poznań, Poland; glysiak@up.poznan.pl

**Keywords:** *Prunus domestica* L., juice, drying technologies, powders, polyphenolics, antioxidant capacity, l-ascorbic acid, hydroxymethylfurfural

## Abstract

Among popular crops, plum (*Prunus domestica* L.) has received special attention due to its health-promoting properties. The seasonality of this fruit makes it impossible to consume it throughout the year, so new products in a powder form may offer an alternative to fresh consumption and may be used as high-quality natural food ingredients. A 100% plum (cultivar “Valor”) juice was mixed with three different concentrations of maltodextrin or subjected to sugars removal by amberlite-XAD column, and dried using the freeze, spray, and vacuum (40, 60, and 80 °C) drying techniques. The identification and quantification of phenolic acids, flavonols, and anthocyanins in plum powders was performed by LC-MS QTof and UPLC-PDA, respectively. l-ascorbic acid, hydroxymethylfurfural, and antioxidant capacity were measured by the Trolox equivalent antioxidant capacity (TEAC) ABTS and ferric reducing antioxidant potential (FRAP) methods in order to compare the influence of the drying methods on product quality. The results indicated that the profile of polyphenolic compounds in the plum juice powders significantly differed from the whole plum powders. The drying of a sugar free plum extract resulted in higher content of polyphenolic compounds, l-ascorbic acid and antioxidant capacity, but lower content of hydroxymethylfurfural, regardless of drying method applied. Thus, the formulation of plum juice before drying and the drying method should be carefully selected in order to obtain high-quality powders.

## 1. Introduction

European plum (*Prunus domestica* L.) is a popular species cultivated in the European Union on an area of over 190 thousand hectares. With a total crop of 1.6 million tonnes, the EU production represents about 15% of the world’s production of this fruit [1]. Plum cultivars differ in terms of chemical composition [2], and European plum cultivars intended for prune production [3], including “Valor”, have been found to be rich in bioactive compounds [4]. The plum is a seasonal fruit, which means that it cannot be consumed fresh throughout the year, so designing new products in a powdered form may offer an attractive alternative. Various processed commodities have been extensively examined for their potential benefits for human health, including for their ability to diminish bone loss [5,6], reduce the risk of cardiometabolic disorders [7], and prevent the aging processes [8]. The main polyphenolic compounds present in plums are phenolic acids, such as chlorogenic acids, anthocyanins, flavanols, flavonols, and coumarins [9,10,11], which have been found to have numerous pharmacological effects, including antioxidant activity, anticancer, antimutagenic, and anti-inflammatory properties [11]. Due to the presence of those valuable, biologically active compounds in plums, plum processing seems to be of particular relevance to preserving those compounds in plum products available on the market throughout the year, including products offered in a powdered form [4]. Powder is the most common form of food additives, as it is the most stable form of natural products that is easy to use and simple to pack, distribute, and handle [12]. Fruit powder products can be easily applied in numerous foodstuffs, and even pharmaceutical products, to improve their colour and flavour [13] and, at the same time, to provide the human body with additional health-related constituents. The drying techniques used in industrial fruit processing to produce fruit powders include, apart from conventional air drying, freeze drying, vacuum drying, and spray drying [14]. The application of vacuums in drying systems allows the reduction of the drying temperature. Thus, it makes it possible to improve the product quality in terms of the better retention of aroma and nutrient compounds in the final products when compared to conventional drying [15]. Among the drying techniques currently used to obtain powders from fruit products, freeze drying is considered to be the best method because the low-temperatures applied in this process allow the highest retention of bioactive compounds [16]. The major drawback of freeze drying lies in a low drying rate and the application of refrigeration and vacuum systems, which, taken together, result in high energy consumption [17]. A very effective method in which liquid products are directly turned into fine powder is spray drying. This process is applied broadly in industry, especially for processing diary and fruit products [18]. This technique was proved to be four to five times cheaper when compared to freeze drying [19]. Because of the Maillard reaction, drying processes and parameters applied to fruit powdering might noticeably alter the quality of products in terms of the presence and quantity of the biologically active components. Factors influencing the formation of the Maillard reaction and caramelization products include time, temperature, water activity, pH, and substrate concentration [20]. Vvedenskaya et al. [21] showed that freeze-dried cranberry powder contained flavonol glycosides that were not identified in the fresh fruit. Furthermore, Michalska et al. [4] showed that the drying of whole plums by different drying techniques resulted in the degradation of major polyphenolic compounds (chlorogenic acids); however, the application of microwave vacuum drying with a magnetron power of 480 W at above 120 °C (the highest temperature applied in this method during the experiment) led to an increase in the content of 4-*p*-coumaroylquinic acid in the obtained powders. The profile and level of bioactive compounds as well as the physical properties of final products might be also affected by the formulation of the material subjected to drying. Fruit powders can be obtained from whole fruit [4], fruit by-products [22,23], or juices/concentrates [24,25]. Of the three listed types of input material, juices and concentrates present a particular challenge to food engineers. This is because a number of fruit juices cannot be simply converted to powder due to their inherent composition, especially due to the presence of low molecular weight acids and carbohydrates that have a low glass transition temperature [25], and stickiness behaviour. These difficulties can be overcome by adding to the input material before drying a carrier agent, such as biopolymers from different sources (maltodextrins with different dextrose equivalence, natural gums, proteins, waxes), that will affect the physicochemical properties of the final powders [26]. The formulation of fruit material before drying and the drying processes themselves might provoke positive and negative changes in the composition of fruit powders. Therefore, the aim of this study was to evaluate the effect of the plum juice formulation (such as the addition of a carrier or the removal of sugars) and different drying methods on the profile and quantity of polyphenolic compounds, vitamin C, hydroxymethylfurfural in, and the antioxidant capacity of, plum juice powders.

## 2. Results and Discussion

### 2.1. Polyphenolic Compounds

The identification and quantification of polyphenolic compounds present in the plum juice powders obtained by different drying techniques was performed by LC-MS QTof according to UV/VIS spectra, MS, MS/MS, molecular ions [M − H]^−^ and [M + H]^+^ (Table 1), and UPLC-PDA (Table 2). The profile and quantity of the polyphenolic compounds present in the plum juice powders differed in comparison to powders obtained from whole plums (by drying of plum halves) [4]. In the current study, 12 polyphenolic compounds were identified: five phenolic acids, four flavonols, and three anthocyanins (Table 1 and Table 2). Their total content ranged from 0.03 to 4.8 g·kg^−1^ dry basis (db) of plum juice powder and was significantly lower when compared to the total polyphenol content in the whole plum powders obtained by different drying techniques (7.8–14.9 g·kg^−1^ db) [4]. This might be due to the fact that none of the compounds that were identified in the plum juice powders belong to flavan-3-ols, which were found in the whole plum powders [4].

Similar to previous studies [4,11,27], the major group of polyphenolic compounds (≈77% of all polyphenols) consisted of polyphenolic acids, and their composition varied from those present in the whole plum powders, as no 4-*p*-coumaroylquinic and caffeoylshikimic acids were identified in the plum juice powders. Contrary to plum dried products [4,11,27] in which chlorogenic and neochlorogenic acids were the dominant constituents, 3-feruloylquinic acid was the major phenolic acid present in the plum juice powders, thus indicating a strong influence of fruit fractions present in the juice on the final composition of the powders.

The juice composition (sugar free juice extract vs. juice with 15%, 25%, and 35% maltodextrin content) influenced the content of polyphenolic acids. The highest level of polyphenolic acids was noted in powders obtained from the sugar free juice extract—it was approximately 78 times higher when compared to their average content in the juice powders containing maltodextrin. The addition of maltodextrin to the juice made the matrix of powders more complex, leading to the probable interaction between the polyphenolic compounds and those compounds and carriers that generally lowered the content of polyphenolic acids in the powders. Regardless of juice formulation, the drying method and drying parameters had an influence on the content of individual polyphenolic acids. Basically, freeze drying (FD) and spray drying (SD) resulted in comparable amounts of phenolic acids in the powders, except methyl-3-caffeoylquinate. Methyl-3-caffeoylquinate, previously identified only in fresh plums of selected cultivars [11], but present neither in dried plums [27] nor in whole plum powders [4], increased 5, 8, and 12 times in the plum juice powders with 15%, 25%, and 35% maltodextrin content, respectively, along with the rise in temperature from 40 up to 80 °C during vacuum drying (VD) (Table 2). In the case of the powders obtained from the sugar free juice extract, a 20 times increase in the content of this constituent within the above-mentioned temperature range was noted. What is more, a significantly stronger (up to 16 times) increase in the content of methyl-3-caffeoylquinate was noted after SD as compared to FD, regardless of juice formulation before dehydration. This might suggest that the release of methyl-3-caffeoylquinate from other bounded forms was accelerated by the high drying temperature. Thus, as it was previously indicated for other fruit juice powders [24], this drying method might be considered as representing a competitive alternative to FD in the production of plum juice powders.

It was noted that in the case of powders obtained from the juice formulation containing 35% maltodextrin, no noticeable changes in the content of 3-feruloylquinic acid, neochlorogenic acid, and 3-*O*-*p*-coumaroylquinic acid were noted. It might be concluded that the addition of this carrier at a sufficiently high concentration might have a protective effect on the aforementioned compounds during the drying of the plum juice.

The second group of phenolic compounds (≈21% of all polyphenols) present in the plum juice powders consisted of four flavonols (Table 1). The flavonols identified in the plum juice powders, and their quantity, which ranged from 6 to 991 mg·kg^−1^ db of powder, significantly differed from the flavonols and quantities identified in the whole plum powders [4]. It was indicated in one of the previous studies that this group of polyphenols was present in the smallest quantity (up to 3% of all polyphenolic compounds) and ranged from 207 to 498 mg·kg^−1^ db of powder [4]. In the current study, the dominant flavonols were: quercetin-3-*O*-galactoside and quercetin-3-*O*-glucoside (Table 2), whereas quercetin-3-*O*-xyloside, which was the predominant flavonol in the whole plum powders [4] was not detected. Quercetin-3-*O*-(6′′acetyl-galactoside) was for the first time identified in the plum products (Table 1).

Similar to phenolic acids, the juice formulation before drying noticeably affected the content of individual flavonols in the obtained powders. The increased concentration of maltodextrin lowered the content of individual flavonols, whereas the removal of sugars from the plum juice before drying resulted in approximately 82 times higher content of flavonols in the powders when compared to the average value determined in the plum juice powders obtained from the juice containing maltodextrin. The content of quercetin-3-*O*-glucoside, regardless of juice formulation, was similarly affected by the drying methods applied in the study, except FD. The latter process noticeably diminished the quantity of quercetin-3-*O*-glucoside when compared to VD and SD, but only in the powders obtained from the juice with the addition of 25% and 35% maltodextrin. This probably resulted from the hindered release of this constituent from the matrix formed from the combination of juice and maltodextrin during the extraction procedure of freeze dried products. A strong degradation of this constituent was previously noted in the whole plum powders after FD [4]. As for quercetin-3-*O*-galactoside, VD at 80 °C resulted in an, on average, approximately 10 times greater reduction in the content of this compound in the powders with maltodextrin, and a 3.4 times greater reduction in the powders obtained from the sugar free juice extract as compared to FD. Contrary to the content of quercetin-3-*O*-glucoside, a relatively low content of quercetin-3-*O*-galactoside was noted after SD, suggesting that the level of this compound was strongly affected by the drying temperature. In the case of quercetin-3-*O*-rutinoside and quercetin-3-*O*-(6′′acetyl galactoside), the increase in the drying temperature resulted in the interconversion of these quercetin conjugates. An average increase of approximately 95% was noted after VD at 80 °C when compared to VD at 40 °C (Table 2), which might suggest that the higher the temperature is during drying, the larger the amounts of this constituent that are released from bounded forms and the faster the release.

Previously, six main anthocyanins were identified in fresh plums [9,27]. In the current study, similar to Michalska et al. [4], two cyanidins and one peonidin which constituted ≈2% of all phenolic compounds were detected in the powders obtained from the sugar free juice extract. At the same time, no peonidin-3-*O*-rutinoside was detected in the powders prepared from the juice with the addition of maltodextrin.

Among the identified anthocyanins, cyanidin-3-*O*-rutinoside was the major compound making up approximately 70% of all anthocyanins present in the analysed powders. This was in agreement with the data regarding fresh plums [28] and whole plum powders [4]. The content of anthocyanins was influenced by the juice formulation, as the total content of those compounds in the powders obtained from the sugar free juice extract was approximately 130 times higher than their average content in the powders obtained from the juice with the addition of maltodextrin. The quantity of those constituents in the powders obtained from the sugar free juice extract was comparable to that in the whole plum powders obtained after convective drying [4]. Dehydration noticeably influenced the levels of individual anthocyanins in the obtained powders and, as was previously stated, the high temperature and the time of the processes were the major factors responsible for their degradation [27,29]. Among the drying techniques applied, SD resulted in the highest retention of anthocyanins, even when compared to FD, which was considered to be the best method for obtaining high-quality dehydrated food products [16].

### 2.2. l-Ascorbic Acid (AA)

The average content of AA in fresh plums was previously estimated at the level of 9.5 mg·100 g^−1^ [30] and differed depending on the plum cultivar [10]. Significant differences in the content of this constituent in plum flesh and skin have previously been identified [10]. Drying processes significantly affected the content of ascorbic acid as a noticeable decrease, even down to 80%, in its content was noted after drying at 60 and 85 °C; additionally, the ascorbic acid content was dependent on the cultivar used for drying [27]. In the current study, the content of ascorbic acid ranged from 10 to 34 mg 100 g^−1^ db of plum juice powder. The plum juice formulation affected the content of ascorbic acid in that it was higher by, respectively, 28%, 37%, and 38% in the powders obtained from the sugar free juice extracts when compared to the AA average content in the powders with the addition of, respectively, 15%, 25%, and 35% maltodextrin. As was indicated in a previous study on plum products, the thermal stability of ascorbic acid was strictly connected with the temperature of the processes applied [27]. The content of AA obtained after FD and VD at 40 and 60 °C was similar in all powders regardless of juice formulation (sugar free juice extract; juice with 15%, 25%, and 35% maltodextrin). The application of higher temperatures (i.e., 80 °C during VD and 180 °C during SD) made it possible to obtain the uppermost retention of AA, which was the highest in the case of SD of sugar free juice extracts. As was previously stated, processing under low oxygen with controlled temperature, air velocity, and humidity might decrease the oxidative effect on ascorbic acid and might limit the degradation of ascorbic acid during the drying of plant material [31]. In the present study, all the drying processes were performed under low oxygen conditions. The lower reduction in AA content along with the increase in the processing temperature during VD might have been related to the shorter processing time and to higher humidity in the environment of the samples, as drying at 80 °C was shorter. This indicates that processing time has a stronger impact on AA degradation than drying temperature. As a result, faster water evaporation increased the humidity in the oven and caused less degradation of ascorbic acid than had been previously observed for kiwifruit products [32] and other vegetable products [31]. Thus, the processing conditions significantly influenced the content of ascorbic acid in the final dry products, and the quality of the plum juice powders measured in terms of the content of ascorbic acid might be improved by controlling the time and temperature of drying.

### 2.3. 5-Hydroxymethylfurfural (HMF)

HMF is considered to be a marker of quality for a broad range of processed fruit products [33,34]. Hydroxymethylfurfural is formed as an intermediate compound via the Maillard reaction from direct sugar dehydration at low pH [35] or might be formed as an end product of ascorbic acid decomposition [36]. Previously, HMF was identified in dried plums [27,34] and whole plum powders obtained using different drying technologies [4]. In the current study, HMF was noted in trace amounts close to the detection threshold in all of the analysed samples of powders obtained from the sugar free juice extract (Table 3). The results clearly indicated that the absence of free sugars in the sugar free juice extract influenced the significantly small formation of this compound in the plum juice extract powders, regardless of the drying method applied. On the other hand, such low content of HMF in the powders obtained from the sugar free extract might have resulted from direct hydration of sugars under acidic condition via caramelization reaction [35]. A noticeably higher formation of the HMF was noticed after the drying of the juices containing maltodextrin. It was observed that the increased content of maltodextrin present in the plum juice resulted in lower formation of HMF. This led to the conclusion that maltodextrin might hinder the formation of HMF during the drying of plum juice. The combination of temperature and drying time strongly affected the HMF content in the plum juice powders with maltodextrin. The highest content of this constituent was noted after VD at 80 °C, regardless of maltodextrin content.

Previously, Zhang et al. [37] indicated that in their model system the formation of HMF increased with the increased concentration of chlorogenic acid, especially under low pH. In the current study, no significant correlation between the content of chlorogenic acid and the formation of HMF was noted in the plum juice with the addition of maltodextrin. What is more, during the drying of the sugar free juice extract, a strong negative correlation was observed between the content of HMF and chlorogenic acid (*r* = −0.818). It can be stated that the behaviour of compounds in food products might differ from that assumed in artificial models.

Previously, the decomposition of ascorbic acid was correlated with the formation of HMF during processing, including the storage of different fruit products [27,38]. It was observed that during plum drying, the content of ascorbic acid diminished along with the increase in temperature [27], whereas Del Caro et al. [39] revealed that when different plum cultivars were subjected to dehydration, a higher content of AA was observed after drying at 60 °C when compared to drying at a combination of temperatures (85 and 70 °C). In the current study, the content of ascorbic acid was negatively correlated with HMF (*r* = −0.713) in powders obtained from the sugar free juice extracts, regardless of drying method applied, suggesting its possible part in the formation of HMF. On the other hand, in the powders obtained from the plum juice containing maltodextrin, a strong positive correlation between AA and HMF was indicated (*r* = 0.834), leading to the conclusion that the formation of HMF might be accelerated by the presence of simple sugars in plum juice.

### 2.4. Antioxidant Capacity

In the current study, the formulation of the plum juice affected the antioxidant capacity of the obtained powders as measured by the TEAC ABTS and FRAP methods. A significantly higher ability to scavenge the ABTS^•+^ radical cations and greater FRAP values (by, on average, approximately 97%) were noted for the sugar free juice extract as compared to the juice with the addition of maltodextrin. Based on the average results of different drying methods, it was observed that the higher the addition of maltodextrin, the lower the antioxidant capacity values of the plum juice powders. Previously conducted studies showed that drying processes influenced the antioxidant capacity of the dried products measured by different in vitro methods. Piga et al. [27] found that the temperature applied during plum drying was the major factor causing the increase in the content of compounds able to scavenge DPPH^•^ radicals, as the highest values of those compounds were noted after drying at 85 °C (they were higher compared with those observed after drying at 60 °C). Michalska et al. [4] showed that applying high temperatures during the microwave vacuum drying of plums resulted in an increased antioxidant capacity of the final products. In this study, VD at 80 °C increased the antioxidant capacity of the powders obtained from the juice containing 15%, 25%, and 35% maltodextrin by, respectively, 26.5%, 11.5%, and 1% when compared to the FD powders. It was noted that the increase in the content of maltodextrin diminished the formation of the compounds able to scavenge free radicals.

In regards to the powders obtained from the sugar free juice extract, the decrease in the TEAC ABTS values by 18.2% was noted after VD at 80 °C when compared to FD. This might result from the fact that, due to the absence of sugars in the sugar free extract, no compounds able to scavenge ABTS radical cations and reduce Fe^3+^ could be formed at high drying temperatures. A similar observation was made with regard to the FRAP method. The FRAP values noted after VD at 80 °C of the plum juice with 15%, 25%, and 35% maltodextrin were higher by, respectively, 29%, 8%, and 0.6% when compared to those observed after FD. The FRAP values of the powders obtained from the sugar free extract using VD at 80 °C were 11% lower than those of the FD-dried powders.

The increase in the antioxidant capacity and FRAP values in the plum juice powders with the addition of maltodextrin might have resulted from the presence of naturally occurring bioactive components and/or newly formed compounds via Maillard reaction/caramelization. A positive correlation was found between total polyphenol content and TEAC ABTS (*r* = 0.978) and FRAP (*r* = 0.985). The correlation between TEAC ABTS and FRAP and the content of ascorbic acid was *r* = 0.5. What is more, a positive correlation between antioxidant capacity and reducing power and HMF was indicated (*r* = 0.61), suggesting that the formation of HMF might have influenced the overall antioxidant capacity of the powders.

The decrease in the TEAC ABTS and FRAP values in the powders obtained from the sugar free extract using VD at 80 °C might have been attributable to the fact that the naturally occurring components have more impact on the antioxidant properties of the dried product and the absence of sugars in the sugar free extract did not result in the formation of the compounds able to scavenge ABTS radical cations and reduce the Fe^3+^ to Fe^2+^ at high temperatures, as the correlation coefficient was *r* = 0.4 for both methods.

## 3. Material and Methods

### 3.1. Reagents

The 2,2′-azino-bis(3-ethylbenzothiazoline-6-sulfonic acid) diammonium salt, hydroxymethylfurfural, potassium persulfate, 2,4,6-tris(2-pyridyl)-s-triazine, and Trolox^®^ were purchased from Sigma-Aldrich (Buchs, Switzerland). Maltodextrin and Amberlite XAD-16 resin were supplied by Brenntag (Kędzierzyn-Koźle, Poland). Cyanidin-3-*O*-glucoside, peonidin-3-*O*-rutinoside, quercetin-3-*O*-glucoside, quercetin-3-*O*-rutinoside, *p*-coumaric, and ferulic acid were obtained from Extrasynthese (Lyon, France). Chlorogenic and neochlorogenic acids were from TRANS MIT GmbH (Giessen, Germany). Acetonitrile for UPLC (gradient grade) and ascorbic acid were from Merck (Darmstadt, Germany). UPLC grade water prepared using the HLP SMART 1000s system (Hydrolab, Gdańsk, Poland) was additionally filtrated through a PTFE 0.22 µm membrane filter (Millex Samplicity Filter, Merck, Darmstadt, Germany) before use.

### 3.2. Material

Plums (cv. “Valor”) were collected in 2014 from a commercial orchard managed according to the integrated production rules, located at the Research Station of the Poznan University of Life Sciences (Poznan, Poland). The plums (20 kg) were washed, pitted, and mixed using a Thermomix (Wuppertal, Vorkwek, Germany). The mash obtained in this way was pressed by a laboratory hydraulic press (SRSE, Warsaw, Poland) and centrifuged for 10 min at room temperature at 5000× *g* (Sigma 6K15, Shrewsbury, UK). The supernatant of the plum juice of 14.9 °Bx (5 L) was portioned into two parts: (i) from one part (2.5 L) sugar was removed in order to obtain a polyphenol extract. This was done by loading the juice into a glass column filled with amberlite XAD-16 resin previously washed with water [40]. The absorbed compounds were eluted with ethanol and the solvent was removed by a scale rotary evaporator Laborota 20 (Heidolph, Schwabach, Germany) at 40 °C thus producing a sugar free plum juice extract; (ii) the second part (2.5 L) was mixed with 15%, 25%, and 35% (*w*/*w*) commercial maltodextrin (dextrose equivalent: 20–30). Both parts of the juice (the sugar free juice extract and the juices with different maltodextrin contents) were subjected to drying techniques.

### 3.3. Drying Methods

Freeze drying (FD) of the sugar free juice extract and the plum juice with maltodextrin (15%, 25%, and 35%) (≈100 mL) was carried out in a freeze dryer OE-950 (Labor, MIM, Budapest, Hungary) for 24 h at a reduced pressure of 65 Pa. The temperature in the drying chamber was −60 °C, while the heating plate reached 30 °C. The freeze-dried sample was considered to be the control sample.

Vacuum drying (VD) was performed in a vacuum dryer (SPT-200, ZEAMiL Horyzont, Kraków, Poland). The sugar free juice extract and the plum juices with different maltodextrin contents (15%, 25%, and 35%) (≈50 mL) were placed in the drying chamber and dried at three different temperatures: 40, 60 and 80 °C at a pressure of 300 Pa for, respectively, 20, 16 and 10 h.

The resultant alternates of plum juice (≈100 mL) were dried with a mini spray dryer B190 (Buchi, Flawil, Switzerland) (SD). The spray dryer was operated at an inlet temperature of 180 °C and an outlet temperature of 70 °C. The temperature of the plum juice loaded into the spray dryer was 23 °C and the rate of feeding was 400 mL·min^−1^.

All drying techniques were performed at least in duplicate for each variant of plum juice (sugar free juice extract and juice with the addition of 15%, 25%, and 35% maltodextrin). The obtained plum powders (FD, VD, SD) were milled (MKM 6003c, Bosch, Bosch GmbH, Stuttgart, Germany), vacuum packed (PP-5.14, Tepro SA, Koszalin, Poland), and stored at −20 °C until they were analysed.

### 3.4. Moisture Content

The moisture content of plum powders was determined by the vacuum-oven method [41], where the samples were dried for 24 h at 80 °C and 300 Pa. The results (*n* = 3) were expressed as % of dry basis (db).

### 3.5. Identification of Polyphenols by the LC-MS QTof Method

Approx. 0.5–0.06 g of each plum powder was resolubilized with 5 mL of 30% MeOH with 1% ascorbic acid (*v*/*v*) by using a vortex (2 min) and was subjected to sonication for 5 min. The procedure was repeated after 24 h, as previously described [42], and the resultant extracts were centrifuged for 10 min at 15,000× *g* at 4 °C. The presence of polyphenols in plum powders was identified using an Acquity Ultraperformance LC system [42]. The data obtained from LC-MS QTof were assessed using the MassLynx 4.0 ChromaLynx Application Manager software.

### 3.6. Quantification of Polyphenols Using the UPLC-PDA System

Polyphenolic compounds were analysed using the Acquity UPLC system (Waters Corp., Milford, MA, USA) on a BEH C_18_ analytical column (2.1 mm × 100 mm; 1.7 µm) with the flow rate of 0.42 mL·min^−1^. Acetonitrile (100%) was used as a strong wash solvent and acetonitrile−water (10%) as a weak wash solvent. The analytical column was kept at 30 °C by a column oven, whereas the samples were kept at 4 °C. The mobile phase was composed of solvent A (4.5% formic acid) and solvent B (acetonitrile). The elution was as follows: 0−10 min, linear gradient from 1 to 25% B; 10.0−11.5 min, linear gradient from 25% to 100%; 11.5−12.5 min, column washing; and reconditioning for 2.5 min. Photodiode array (PDA) spectra were measured within the wavelength range of 200 to 600 nm (steps of 2 nm). The runs were monitored at the following wavelengths: phenolic acid at 320 nm and anthocyanins at 520 nm. Retention times (*R*_t_) and spectra were compared with those of pure standards. Calibration curves at concentrations ranging from 0.05 to 5 mg·mL^−1^ (*r*^2^ ≤ 0.9998) were made from chlorogenic, neochlorogenic, ferulic acid, cyanidin-3-*O*-glucoside, and cyanidin-3-*O*-rutinoside as standards. Feruloylquinic acid was expressed as ferulic acid. All determinations were done in duplicate. Results were expressed as milligrams per kilogram of dry basis of plum juice power (mg·kg^−1^ db).

### 3.7. l-Ascorbic Acid

The content of l-ascorbic acid in the plum powders was determined by titration with 2,6-dichlorophenolindophenol, according to PN-90/A-75101/11 [43]. The results (*n* = 3) were expressed as mg·100 g^−1^ db of plum juice powder and were presented as an average (±SD).

### 3.8. Hydroxymethylfurfural (HMF)

Hydroxymethylfurfural was analysed using the Acquity UPLC system (Waters Corp.) [44] with modifications. HMF was detected at 284 nm and quantified using a standard curve. The results were presented as the average (*n* = 3) mg HMF·kg^−1^ db of plum juice powder.

### 3.9. Trolox Equivalent Antioxidant Capacity (TEAC) and Ferric Reducing Antioxidant Potential (FRAP)

Plum powder extracts were obtained by the sonication (2 × 15 min) of 300 mg of samples in 5 mL of 30% aqueous methanol (*v*/*v*). After being kept for 24 h at 4 °C in the dark, the extracts were centrifuged (1500× *g*; 10 min; 4 °C). The antioxidant capacity of the plum powder extracts was determined using the Trolox Equivalent Antioxidant Capacity tests (TEAC ABTS) according to Re et al. [45]. The ferric reducing ability of the extracts was analysed by FRAP assay [46]. All measurements and analyses were performed in triplicate (*n* = 3) and the results were presented as an average (±SD). The data were expressed as mmol Trolox·100 g^−1^ db of plum juice powder.

### 3.10. Statistical Analysis

Statistical analysis was made by Statistica 10 (Statistica, Tulsa, OK, USA). Average values were subjected to one–way analysis of variance (ANOVA) and the least significance test HSD Tukey was used to compare the differences that were found to be significant at *p* < 0.05. In order to investigate the relationship between selected variables, the Pearson correlation coefficient was calculated.

## 4. Conclusions

The formulation of the juices before drying noticeably affected the profile and content of polyphenolic compounds in the obtained powders. The major group of polyphenols present in the plum juice powders consisted of five polyphenolic acids among which 3-feruloylquinic acid was the dominant one. Four flavonols were detected in the plum juice powders. Anthocyanins made up approximately 2% of all polyphenols present in the plum juice powders. In general, among the drying techniques applied, spray drying resulted in the highest retention of polyphenols in plum juice powders, regardless of juice formulation, when compared to freeze drying, which is considered to be the best method for obtaining high-quality dehydrated food products. The processing conditions significantly influenced the content of ascorbic acid in the dried final products as the shorter processing time during vacuum drying at 80 °C resulted in a higher content of ascorbic acid in the plum juice powders. A significantly higher formation of hydroxymethylfurfural was noted in the powders obtained from the plum juice containing maltodextrin when compared to powders obtained from the sugar free juice extract; however, the higher the content of maltodextrin, the lower concentration of HMF in the products. What is more, the determination of hydroxymethylfurfural might be a good tool for monitoring the highly processed plum powders. The antioxidant capacity measured by the TEAC ABTS and FRAP methods was significantly higher in the powders obtained from the sugar free juice extract than in the powders obtained from the juice with maltodextrin. The powders obtained from the juice with maltodextrin owed their antioxidant potential not only to the naturally occurring compounds, but also to the compounds newly formed via Maillard reaction/caramelization. To sum up, the formulation of plum juice before drying and the drying method should be carefully selected in order to obtain high-quality powders.

## Figures and Tables

**Table 1:** Phenolic compounds identified by LC-MS QTof in juice plum powders.

Compound	*R*_t_ (min)	*λ*_max_ (nm)	MS [M − H]^−^ (*m/z*)	MS/MS [M − H]^−^ (*m/z*)
Phenolic acids				
Neochlorogenic acid	3.09	326	353.08	191.05
3-*O-p*-coumaroylquinic acid	3.86	312	337.09	163.05
Chlorogenic acid	4.16	326	353.08	191.05
3-Feruloylquinic acid	4.30	324	367.10	193.05
Methyl-3-caffeoylquinate	4.74	326	367.10	135.00/193.01
Flavonols				
Quercetin-3-*O*-rutinoside	6.50	264/350	609.14	301.03
Quercetin-3-*O*-galactoside	6.66	346	463.08	301.02
Quercetin-3-*O*-glucoside	6.75	346	463.18	301.02
Quercetin-3-*O*-(6′′acetylgalactoside)	8.18	363	505.09	301.02
Anthocyanins				
Cyanidin-3-*O*-glucoside	3.72	278/515	449.10	287.05
Cyanidin-3-*O*-rutinoside	3.93	279/515	595.16	287.05
Peonidin-3-*O*-rutinoside	4.62	286/517	609.17	301.07

**Table 2:** The content of polyphenolic compounds present in plum juice powders obtained by freeze drying (FD), vacuum drying (VD), and spray drying (SD) (mg·kg^−1^ db).

Sample	Drying Method	Phenolic Acids					Flavonols				Anthocyanins		
		3-Feruloylquinic Acid	Neochlorogenic Acid	3-*O-p*-Coumaroyl Quinic Acid	Chlorogenic Acid	Methyl 3-Caffeoyl Quinicate	Quercetin-3-*O*-glucoside	Quercetin-3-*O*-galactoside	Quercetin-3-*O*-rutinoside	Quercetin-3-*O*-(6′′Acetyl galactoside)	Cyanidin-3-*O*-glucoside	Cyanidin-3-*O*-rutinoside	Peonidin-3-*O*-rutinoside
15% M	FD	20.92 ± 0.11 ^g^	17.46 ± 0.08 ^g^	6.56 ± 0.06 ^g^	9.65 ± 0.69 ^g^	0.58 ± 0.01 ^a^	8.57 ± 0.24 ^g^	8.51 ± 0.26 ^j^	2.04 ± 0.21 ^f^	1.67 ± 0.02 ^d^	0.21± 0.01 ^d,e^	0.58 ± 0.01 ^f,g^	LOD
	VD 40 °C	21.89 ± 0.15 ^h^	20.32 ± 0.04 ^h^	9.89 ± 0.01 ^i^	10.29 ± 0.37 ^g^	0.77 ± 0.01 ^a^	9.89 ± 0.07 ^h^	2.43 ± 0.01 ^g^	0.11 ± 0.01 ^a^	1.31 ± 0.01 ^c^	0.27 ± 0.01 ^f^	0.72 ± 0.03 ^h^	LOD
	VD 60 °C	15.91 ± 0.12 ^f^	15.25 ± 0.23 ^f^	7.49 ± 0.26 ^h^	5.41 ± 0.4 ^b–e^	0.71 ± 0.03 ^a^	7.55 ± 0.09 ^e^	1.84 ± 0.02 ^f^	0.42 ± 0.03 ^b^	1.35 ± 0.03 ^c^	0.11 ± 0.02 ^b^	0.31 ± 0.01 ^c,d^	LOD
	VD 80 °C	8.75 ± 0.27 ^a^	11.76 ± 0.05 ^d^	5.11 ± 0.04 ^e^	3.96 ± 0.05 ^a,b^	3.81 ± 0.15 ^c,d^	7.62 ± 0.02 ^e^	0.74 ± 0.02 ^b–d^	2.48 ± 0.02 ^g^	2.84 ± 0.06 ^f^	0.03 ± 0.01 ^a^	0.13 ± 0.02 ^a^	LOD
	SD	21.46 ± 0.11 ^g,h^	17.93 ± 0.19 ^g^	5.74 ± 0.11 ^f^	9.41 ± 0.1 ^g^	5.98 ± 0.36 ^e^	8.22 ± 0.02 ^f^	0.87 ± 0.05 ^c,d^	2.34 ± 0.05 ^d^	1.29 ± 0.02 ^c^	0.39 ± 0.02 ^g^	0.99 ± 0.02 ^i^	LOD
25% M	FD	14.46 ± 0.08 ^e^	11.45 ± 0.33 ^d^	5.74 ± 0.17 ^f^	6.39 ± 0.43 ^e,f^	0.31 ± 0.08 ^a^	0.88 ± 0.01 ^a^	6.03 ± 0.03 ^i^	1.48 ± 0.02 ^d^	1.11 ± 0.01 ^b^	0.28 ± 0.01 ^f^	0.68 ± 0.01 ^h^	LOD
	VD 40 °C	13.52 ± 0.07 ^d^	11.71 ± 0.01 ^d^	5.91 ± 0.05 ^f^	6.11 ± 0.41 ^d–f^	0.43 ± 0.03 ^a^	5.93 ± 0.04 ^d^	1.46 ± 0.02 ^e^	0.04 ± 0.01 ^a^	1.08 ± 0.01 ^b^	0.24 ± 0.01 ^e,f^	0.61 ± 0.02 ^g^	LOD
	VD 60 °C	13.51 ± 0.05 ^d^	11.83 ± 0.09 ^d^	5.31 ± 0.02 ^e^	6.46 ± 0.04 ^e,f^	0.52 ± 0.01 ^a^	5.84 ± 0.02 ^d^	1.43 ± 0.01 ^e^	0.21 ± 0.02 ^a,b^	1.06 ± 0.01 ^b^	0.11 ± 0.03 ^b^	0.29 ± 0.01 ^b,c^	LOD
	VD 80 °C	10.65 ± 0.18 ^b^	10.62 ± 0.11 ^c^	3.92 ± 0.07 ^b,c^	4.13 ± 0.11 ^a–c^	3.33 ± 0.09 ^b,c^	5.28 ± 0.07 ^c^	0.48 ± 0.01 ^a,b^	1.53 ± 0.03 ^d,e^	2.57 ± 0.09 ^e^	0.04 ± 0.01 ^a^	0.12 ± 0.01 ^a^	LOD
	SD	15.32 ± 0.49 ^f^	12.87 ± 0.17 ^e^	7.31 ± 0.08 ^h^	6.91 ± 0.56 ^f^	4.42 ± 0.55 ^d^	6.08 ± 0.05 ^d^	0.67 ± 0.01 ^a–c^	1.75 ± 0.01 ^e^	1.07 ± 0.01 ^b^	0.62 ± 0.01 ^h^	1.51 ± 0.03 ^j^	LOD
35% M	FD	10.08 ± 0.03 ^b^	8.04 ± 0.01 ^a^	3.72 ± 0.04 ^b^	4.78 ± 0.12 ^a–d^	0.25 ± 0.01 ^a^	0.64 ± 0.01 ^a^	4.31 ± 0.01 ^h^	1.05 ± 0.01 ^c^	0.76 ± 0.01 ^a^	0.16 ± 0.01 ^c,d^	0.44 ± 0.05 ^e^	LOD
	VD 40 °C	9.25 ± 0.01 ^a^	7.93 ± 0.03 ^a^	3.05 ± 0.04 ^a^	4.09 ± 0.28 ^a–c^	0.23 ± 0.07 ^a^	3.98 ± 0.03 ^b^	1.01 ± 0.01 ^d^	0.03 ± 0.01 ^a^	0.73 ± 0.01 ^a^	0.1 ± 0.01 ^b^	0.26 ± 0.01 ^b,c^	LOD
	VD 60 °C	9.22 ± 0.04 ^a^	8.21 ± 0.05 ^a^	3.08 ± 0.07 ^a^	5.22 ± 0.43 ^c–e^	0.33 ± 0.01 ^a^	4.07 ± 0.01 ^b^	0.98 ± 0.01 ^d^	0.14 ± 0.01 ^a^	0.74 ± 0.02 ^a^	0.08 ± 0.01 ^b^	0.22 ± 0.01 ^b^	LOD
	VD 80 °C	9.30 ± 0.16 ^a^	8.28 ± 0.2 ^a,b^	4.17 ± 0.08 ^c,d^	3.76 ± 0.08 ^a^	2.74 ± 0.16 ^b^	4.07 ± 0.03 ^b^	0.43 ± 0.02 ^a^	1.18 ± 0.05 ^c^	0.77 ± 0.01 ^a^	0.13 ± 0.01 ^b,c^	0.38 ± 0.02 ^d,e^	LOD
	SD	11.44 ± 0.25 ^c^	8.84 ± 0.01 ^b^	4.43 ± 0.02 ^d^	4.51 ± 0.01 ^a–c^	3.27 ± 0.01 ^b,c^	4.12 ± 0.03 ^b^	0.46 ± 0.01 ^a,b^	1.19 ± 0.02 ^c^	0.68 ± 0.01 ^a^	0.21 ± 0.01 ^d,e^	0.52 ± 0.01 ^f^	LOD
Extract	FD	1051.41 ± 6.37 ^C^	1025.23 ± 12.21 ^C^	439.94 ± 8.73 ^A,B^	414.31 ± 1.11 ^B^	24.61 ± 0.87 ^A^	73.06 ± 1.12 ^A^	500.52 ± 4.87 ^D^	125.2 ± 0.35 ^B^	102.89 ± 0.15 ^B,C^	26.78 ± 0.4 ^B^	70.31 ± 0.93 ^A,B^	0.58 ± 0.01 ^A,B^
	VD 40 °C	996.32 ± 10.69 ^C^	1017.86 ± 2.09 ^C^	472.78 ± 9.93 ^B^	395.82 ± 0.08 ^B^	36.68 ± 1.43 ^A,B^	524.46 ± 1.91 ^C^	129.35 ± 0.54 ^B^	9.21 ± 0.46 ^A^	104.18 ± 0.37 ^B,C^	25.76±0.25 ^B^	69.62 ± 0.37 ^A,B^	0.58 ± 0.02 ^A,B^
	VD 60 °C	851.27 ± 35.55 ^B^	885.16 ± 10.81 ^B^	458.76 ± 14.99 ^A,B^	358.45 ± 2.46 ^A^	54.74 ± 2.41 ^B^	514.07 ± 6.79 ^C^	141.26 ± 0.57 ^B,C^	24.28 ± 3.87 ^A^	88.89 ± 9.94 ^B^	24.37 ± 3.71 ^A,B^	66.42 ± 10.6 ^A,B^	0.55 ± 0.09 ^A,B^
	VD 80 °C	541.81 ± 15.22 ^A^	645.22 ± 2.48 ^A^	405.2 ± 20.67 ^A^	335.76 ± 12.09 ^A^	708.46 ± 10.24 ^D^	458.41 ± 9.48 ^B^	147.09 ± 5.61 ^C^	108.53 ± 16.46 ^B^	60.57 ± 1.45 ^A^	17.18 ± 2.75 ^A^	52.54 ± 3.13 ^A^	0.44 ± 0.07 ^A^
	SD	1169.09 ± 27.83 ^D^	1104.03 ± 33.02 ^D^	529.99 ± 13.61 ^C^	469.95 ± 4.49 ^C^	392.14 ± 5.64 ^C^	610.68 ± 13.8 ^D^	72.61 ± 0.75 ^A^	188.22 ± 16.59 ^C^	119.82 ± 9.5 ^C^	28.05 ± 0.88 ^B^	78.34 ± 3.56 ^B^	0.65 ± 0.03 ^B^

^a–j^—Different lowercase letters within the column in the group: 15%, 25%, and 35% M indicate significant differences between samples (HSD Tukey test; *p* < 0.05); ^A–D^—different capital letters within the column in the group: “extract” indicate significant differences between samples (HSD Tukey test; *p* < 0.05); M, maltodextrin; LOD, limit of detection.

**Table 3:** The dry matter (%), content of vitamin C (mg·100 g^−1^ db), 5-hydroxymethylfurfural (HMF) (mg·kg^−1^ db), and antioxidant capacity (mmol Trolox·100 g^−1^ db) of plum powders obtained using by freeze drying (FD), vacuum drying (VD), and spray drying (SD).

Sample	Drying Method	Dry Matter	Ascorbic Acid	Hydroxymethylfurfural	Antioxidant Capacity	
					TEAC ABTS	FRAP
15% M	FD	93.32 ± 0.17	12.41 ± 0.41 ^a–c^	0.32 ± 0.07 ^a,b^	2.66 ± 0.23 ^c^	2.13 ± 0.06 ^e^
	VD 40 °C	97.20 ± 0.03	9.93 ± 0.72 ^a^	0.77 ± 0.01 ^a,b,c^	3.06 ± 0.01 ^d^	2.75 ± 0.07 ^e^
	VD 60 °C	98.74 ± 0.06	10.63 ± 0.78 ^a^	1.36 ± 0.03 ^c^	2.77 ± 0.09 ^c^	2.28 ± 0.05 ^e^
	VD 80 °C	99.31 ± 0.01	20.23 ± 0.48 ^e^	11.14 ± 0.88 ^e^	3.37 ± 0.02 ^d^	3.01 ± 0.13 ^f^
	SD	97.71 ± 0.01	14.41 ± 1.36 ^b–d^	0.42 ± 0.02 ^a–c^	2.42 ± 0.26 ^c^	2.01 ± 0.16 ^e^
25% M	FD	94.67 ± 0.06	11.41 ± 0.01 ^a,b^	0.05 ± 0.005 ^a^	2.06 ± 0.03 ^b^	1.56 ± 0.07 ^c^
	VD 40 °C	96.20 ± 0.04	10.21 ± 1.29 ^a^	0.12 ± 0.01 ^a^	1.71 ± 0.02 ^a^	1.56 ± 0.1 ^c^
	VD 60 °C	98.61 ± 0.03	11.58 ± 0.13 ^a,b^	0.44 ± 0.02 ^a–c^	2.06 ± 0.1 ^b^	1.53 ± 0.1 ^c^
	VD 80 °C	99.13 ± 0.01	15.37 ± 1.21 ^c,d^	4.05 ± 0.29 ^d^	2.31 ± 0.02 ^b^	1.71 ± 0.06 ^d^
	SD	98.78 ± 0.01	11.25 ± 1.08 ^a,b^	0.48 ± 0.04 ^a–c^	1.81 ± 0.11 ^a^	1.57 ± 0.06 ^c^
35% M	FD	95.62 ± 0.03	11.35 ± 0.06 ^a,b^	0.05 ± 0.01 ^a^	1.63 ± 0.12 ^a^	1.22 ± 0.12 ^b^
	VD 40 °C	95.48 ± 0.01	10.99 ± 0.18 ^a^	0.10 ± 0.01 ^a^	1.47 ± 0.01 ^a^	1.01 ± 0.14 ^a^
	VD 60 °C	98.07 ± 0.10	9.22 ± 0.65 ^a^	0.21 ± 0.005 ^a^	1.45 ± 0.03 ^a^	0.97 ± 0.18 ^a^
	VD 80 °C	98.98 ± 0.03	15.92 ± 0.86 ^d^	1.26 ± 0.19b ^c^	1.65 ± 0.11 ^a^	1.21 ± 0.07 ^b^
	SD	98.98 ± 0.01	10.72 ± 0.65 ^a^	0.41 ± 0.03 ^a,b,c^	1.6 ± 0.13 ^a^	1.07 ± 0.09 ^a^
Extract	FD	92.57 ± 0.14	14.61 ± 1.69 ^A^	0.01 ± 0.001 ^A^	98.9 ± 3.24 ^C^	85.44 ± 4.14 ^C^
	VD 40 °C	96.25 ± 0.17	14.55 ± 0.27 ^A^	0.02 ± 0.001 ^A,B^	95.15 ± 1.33 ^B^	80.92 ± 2.5 ^C^
	VD 60 °C	91.83 ± 1.46	16.08 ± 1.21 ^A^	0.03 ± 0.004 ^C^	98.03 ± 0.26 ^C^	87.15 ± 2.53 ^D^
	VD 80 °C	96.71 ± 0.08	15.28 ± 1.21 ^A^	0.02 ± 0.001 ^A,B^	80.86 ± 0.82 ^A^	75.92 ± 0.78 ^A^
	SD	97.56 ± 0.06	33.78 ± 1.96 ^B^	LOD	81.18 ± 3.84 ^A^	78.3 ± 1.81 ^B^

^a–f^—Different lowercase letters within the column in the group consisted of 15%, 25%, and 35% M indicate significant differences between samples (HSD Tukey test; *p* < 0.05); ^A–D^—different capital letters within the column in the group: “extract” indicate significant differences between samples (HSD Tukey test; *p* < 0.05); db, dry basis; M, maltodextrin; LOD, limit of detection; TEAC ABTS, Trolox Equivalent Antioxidant Capacity; FRAP, Ferric Reducing Antioxidant Potential.
